# Characterization of a phospholipid‐regulated β‐galactosidase from *Akkermansia muciniphila* involved in mucin degradation

**DOI:** 10.1002/mbo3.796

**Published:** 2019-02-06

**Authors:** Konrad Kosciow, Uwe Deppenmeier

**Affiliations:** ^1^ Institute of Microbiology and Biotechnology University of Bonn Bonn Germany

**Keywords:** human gut, intestinal tract, metabolic disorder, microbiota, mucin‐degrading bacteria

## Abstract

The gut microbe *Akkermansia muciniphila* is important for the human health as the occurrence of the organism is inversely correlated with different metabolic disorders. The metabolism of the organism includes the degradation of intestinal mucins. Thus, the gut health‐promoting properties are not immediately obvious and mechanisms of bacteria‐host interactions are mostly unclear. In this study, we characterized a novel extracellular β‐galactosidase (Amuc_1686) with a preference for linkages from the type Galβ1–3GalNAc. Additionally, Amuc_1686 possesses a discoidin‐like domain, which enables the interaction with anionic phospholipids. We detected a strong inhibition by phosphatidylserine, phosphatidylglycerol, phosphatidic acid, and lysophosphatidic acid while phosphatidylcholine and phosphatidylethanolamine had no influence. Amuc_1686 is the first example of a prokaryotic hydrolase that is strongly inhibited by certain phospholipids. These inhibiting phospholipids have important signal functions in immune response and cell clearance processes. Hence, Amuc_1686 might be regulated based on the health status of the large intestine and could therefore contribute to the mutualistic relationship between the microbe and the host on a molecular level. In this sense, Amuc_1686 could act as an altruistic enzyme that does not attack the mucin layer of apoptotic epithelial cells to ensure tissue regeneration, for example, in areas with inflammatory damages.

## 1 INTRODUCTION

*Akkermansia (A.) muciniphila* is a Gram‐negative, anaerobic bacterium, belonging to the phylum Verrucomicrobia (Derrien, Vaughan, Plugge, & Vos, 2004). The organism is specialized in the degradation of highly glycosylated proteins, known as mucins that are found on the surface of epithelial cells in the mammalian gut. Notably the cecum, where the highest amount of mucin is produced, shows the highest numbers of *A. muciniphila*(Derrien et al., 2011). Recent findings about the importance of the composition of the human gut microbiota for individual health led to an increasing interest in *A. muciniphila*. It was found that in most cases the organism has a positive influence on different gut diseases and metabolic disorders (Schneeberger et al., 2015). It was also shown that the presence of *A. muciniphila* is inversely correlated with obesity and type 2 diabetes (Everard et al., 2013). Furthermore, a normalized abundance of the organism, reached by prebiotic feeding, led to an improved metabolic profile (Everard et al., 2013; Shin et al., 2013). In detail, high‐fat diet‐induced disorders as adipose tissue inflammation, fat‐mass gain and insulin resistance were reversed while inflammation control, gut peptide secretion and mucus layer thickness were improved. In addition, the gut barrier function was increased by strengthening the enterocyte monolayer integrity (Everard et al., 2013; Reunanen et al., 2015). Moreover, gastrointestinal disturbance of individuals with autism could be linked with a low relative abundance of *A. muciniphila*(Wang et al., 2011). Interestingly, most of the described effects required viable cells while heat‐killed cells did not improve the metabolic profiles (Everard et al., 2013). Only a few studies suggest a potentially negative effect of *A. muciniphila*at some circumstances, such as an exacerbation of gut inflammation in *Salmonella typhimurium*‐infected gnotobiotic mice or the potentially harmful effect in conditions of a disrupted epithelial barrier (Ganesh, Klopfleisch, Loh, & Blaut, 2013; Kang et al., 2013).

The mucus layer that covers epithelial cells offers many ecological advantages to gut bacteria as carbon and nitrogen source, particularly in the colon where free carbon sources are limited (Derrien, Collado, Ben‐Amor, Salminen, & Vos, 2008; Salyers, West, Vercellotti, & Wilkins, 1977). 16S RNA gene analysis of this complex microbial ecosystem and enrichment cultures of mucin‐degrading bacteria from human feces indicated that *A. muciniphila*is a common and prevalent member of mucin‐utilizing organisms in the human intestinal tract (Collado, Derrien, Isolauri, Vos, & Salminen, 2007; Derrien et al., 2008, 2004). In spite of these obvious benefits that contribute to a decrease of different symptoms associated with metabolic diseases in the mammalian gut, mechanisms of bacteria‐host interactions and exact mucin‐degrading processes of *A. muciniphila*remained mostly unclear (Reunanen et al., 2015). It is known that only a fraction of colonic microbes is able to degrade mucin, providing nutrients also for other members of the colonic microbiota (Derrien et al., 2004; Hoskins & Boulding, 1981). Due to the high complexity and diversity of intestinal mucin glycan structures, cooperative action of different mucin‐degrading organisms and different enzymes as sulfatases, proteases, and especially different glycoside hydrolases (GH) are necessary for an efficient degradation (Crost et al., 2016; Derrien et al., 2004; Willis, Cummings, Neale, & Gibson, 1996). Examples for these important GH family members are fucosidases (GH29 and GH95), exo‐ and endo‐ β‐*N*‐acetylglucosaminidases (GH84 and GH85), neuraminidases/sialidases (GH33), endo‐ β 1–4‐galactosidases (GH98), and β ‐galactosidases (GH2, GH20 and GH42) (Crost et al., 2016).

In this study, a new type of β‐galactosidase from *A. muciniphila*is described which belongs to GH family GH35. We were able to show a strong inhibitory effect of anionic phospholipids on this β‐galactosidase, which might indicate a regulatory function of the C‐terminal domain and is, to our knowledge, the first characterization of a prokaryotic hydrolase that is strongly inhibited by certain phospholipids. During the degradation of intestinal mucins, this regulatory module could be responsible for the discrimination between apoptotic and non‐apoptotic epithelial cells. In this case, the C‐terminal domain might increase the specificity of the extracellular hydrolase for epithelial mucins of viable cells.

## 2 EXPERIMENTAL PROCEDURES

### 2.1 Materials

All chemicals, reagents and substrates used in this study were purchased from Carl Roth GmbH (Karlsruhe, Germany), Sigma‐Aldrich (Munich, Germany), or Megazyme (Bray, Ireland). *Taq*DNA polymerase, T4 ligase, restriction endonucleases and PCR reagents were obtained from Fermentas (St. Leon‐Rot, Germany) and New England Biolabs (Frankfurt am Main, Germany). Oligonucleotides were synthesized by Eurofins (Ebersberg, Germany).

### 2.2 Culture conditions and standard molecular techniques

*Escherichia coli*DH5α and *E. coli*BL21 (DE3) were grown in lysogeny broth (Miller, 1972). For plasmid maintenance µg ml^‐1^ ampicillin was added. All standard molecular techniques used in this study were done according to Sambrook, Fritsch, and Maniatis (1989).

### 2.3 Construction of an amuc_1686 expression system

The β‐galactosidase from *A. muciniphila*encoded by *amuc_1686*was amplified via PCR without its native signal sequence and with or without its C‐terminal discoidin domain. Each PCR product contained the endonuclease restriction sites *SnaB*I and *Xho*I at the 5′and 3′ end respectively. The fragments were ligated into the corresponding restriction sites of pASK‐IBA5 resulting in the expressions vectors pASK‐IBA5_noSP‐amuc_1686 and pASK‐IBA5_noSP‐amuc_1686_short (Table 1).

**Table 1:** Strains, plasmids, and primers

Strain, plasmid, primer	Description or sequence	Source or restriction site (underlined)
Strains		
*Escherichia coli*DH5α	*fhuA2 Δ(argF‐lacZ)U169 phoA glnV44 Φ80 Δ(lacZ)M15 gyrA96 recA1 relA1 endA1 thi‐1 hsdR17*	New England Biolabs, Frankfurt am Main, Germany
*Escherichia coli*BL21	*fhuA2 [lon] ompT gal (λ DE3) [dcm] ∆hsdS* *λ DE3 = λ sBamHIo ∆EcoRI‐B int::(lacI::PlacUV5::T7 gene1) i21 ∆nin5*	New England Biolabs, Frankfurt am Main, Germany
Plasmids		
pASK‐IBA5	Vector carrying an inducible tetracycline promotor/operator, ampicillin resistance cassette, f1 origin, MCS and Strep‐tag for *N*‐terminal fusion to the recombinant protein	IBA, Göttingen, Germany
pASK‐IBA5_noSP‐amuc_1686	pASK‐IBA5 derivative containing *amuc_1686*from *Akkermansia muciniphila*ATCC BAA‐835 without its native signal peptide	This study
pASK‐IBA5_noSP‐amuc_1686_short	pASK‐IBA5 derivative encoding *amuc_1686*from *Akkermansia muciniphila*ATCC BAA‐835 without its native signal peptide and without C‐terminal discoidin domain	This study
Primer		
pASK5_natSP‐amuc_1686.fw	ATTAGAGCTCTAAATTATCCTTT	*Sac*I
pASK5_noSP‐amuc_1686.fw	ATTAGAGCTCTGCTGCTCCCATGCCTTT	*Sac*I
pASK5_amuc_1686.rev	ATTACTCGAGTTACTTGGCAGGCTTGAAC	*Xho*I
pASK5_amuc_1686‐short.rev	ATTACTCGAGTTAATGGGGGCCGTTGTCA	*Xho*I

### 2.4 Overexpression and purification of Amuc_1686 and Amuc_1686_short

*Escherichia coli*DH5α cells were used for vector cloning and vector amplification. For protein production overnight cultures of *E. coli*BL21 DE3 (5 ml) harboring plasmids of interest were used to inoculate 1 L LB medium and were incubated at 37°C in shaker flasks at 180 rpm. When the cultures reached an optical density at 600 nm of approximately 0.4, protein production was induced by addition of 0.2 µg ml^‐1^ anhydrotetracycline. Cells were harvested at an optical density between 1.0 and 1.5 by centrifugation at 9,000 *g*. Lysis and purification was done as previously described (Kosciow, Domin, Schweiger, & Deppenmeier, 2016). Polyacrylamide gel electrophoresis was performed according to Laemmli (1970) and visualization of the protein bands via silver stain was done as described by Blum, Beier, and Gross, 1987. Native conformation of Amuc_1686 was analyzed by applying the protein to a gel filtration chromatography using a HiLoad 16/60 Superdex 75 pg column (GE Healthcare) connected to an ÄKTApurifier system (GE Healthcare). Equilibration was done with 50 mM Tris‐HCl buffer pH 7, containing 150 mM NaCl.

### 2.5 Measurement of enzyme activities

Enzyme activity assays for Amuc_1686 were performed in a combined buffer system containing sodium acetate, Tris‐HCl, potassium dihydrogen phosphate and dipotassium phosphate (50 mM each). In case of colorimetric *p*‐nitrophenol substrates, the activity was measured photometrically at 420 nm. The optimum pH of Amuc_1686 was determined using a combined buffer in the range from pH 5 to 10 at 30°C. The temperature optimum was determined at optimum pH and temperatures ranging from 20 to 80°C. Enzyme kinetics were measured by varying substrate concentration (0–40 mM) at optimal pH (7.5) and temperature (65°C). Inhibition experiments were performed at 37°C and a 50 mM combined buffer system pH 7.5 was used. All potentially inhibiting compounds were dissolved in methanol prior to assay application. The impact of different ions on the enzyme activity was analyzed in 100 mM Tris‐HCl buffer pH 7.5.

### 2.6 Determination of products from non‐colorimetric substrates

For substrate spectrum analysis with non‐colorimetric substrates, enzymatic assays were performed as described above and incubated for different time periods at 37°C. Samples were applied to HPLC using an Aminex‐HPX87H column (BioRad, 300 × 7.8 mm) with 5 mM H_2_SO_4_as mobile phase at 25°C and a flow rate of 0.6 ml min^‐1^. The amounts of products and the grade of substrate degradation was determined, using an UV‐Vis detector at 210 nm and a refractive index detector. Products were quantified by comparison to calibration curves.

## 3 RESULTS

### 3.1 Characterization of Amuc_1686

In this study, we identified and characterized the hypothetical GH Amuc_1686 from the anaerobic, mucin‐degrading gut microbe *A. muciniphila* ATCC BAA‐835. The corresponding gene *amuc_1686*encodes a protein with a molecular mass of 86 kDa. BLAST analysis of Amuc_1686 using the SwissProt database indicated that 19 of the 20 closest hits were exclusively eukaryotic β‐galactosidases from mainly mammalian species such as *Homo sapiens*(Q8IW92), *Mus musculus*(Q3UPY5), and *Rattus norvegicus*(Q5XIL5). Only one protein with significant homology (identity of 44%, query coverage of 77%) was found in the bacterial kingdom, the β‐galactosidase Bga from *Xanthomonas manihotis*(P48982) (Taron, Benner, Hornstra, & Guthrie, 1995). A bioinformatic analysis of the amino acid sequence of Amuc_1686 with SignalP 4.1 (Nielsen, Engelbrecht, Brunak, & Heijne, 1997; Petersen, Brunak, Heijne, & Nielsen, 2011) and Phobius (Käll, Krogh, & Sonnhammer, 2004) revealed a potential signal sequence and a predicted signal peptidase cleavage site between position 17 and 18 at the *N*‐terminus of the protein.

**Figure 1:**
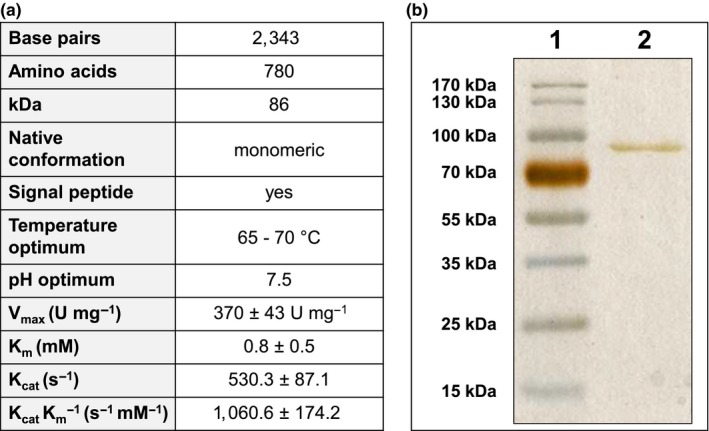
Characterization of purified Amuc_1686. (a) Genetic, structural and biochemical features of purified Amuc_1686. Enzymatic activities were determined with *para‐*nitrophenyl‐β‐d‐galactopyranoside as Substrate. (b) Visualization of Amuc_1686 via polyacrylamide gel electrophoresis and silver stain. Line 1: 3 µg of Amuc_1686. Line 2: Ladder

For heterologous protein production in *E. coli*BL21, the gene *amuc_1686*without its native predicted signal sequence was amplified via PCR and ligated into pASK‐IBA5, which allowed an *N*‐terminal addition of a Strep‐tag sequence to the protein. Amuc_1686 was purified by streptavidin affinity chromatography to apparent homogeneity. A total of 2.5 mg of protein was obtained from 1 L *E. coli* culture. The enzyme showed a single band at 87 kDa when analyzed by polyacrylamide gel electrophoresis and silver stain which was in agreement with the predicted size of the recombinant tagged protein without signal peptide (Figure 1a,b).

Gel filtration chromatography revealed a monomeric native structure for Amuc_1686. Purified Amuc_1686 showed the highest catalytic efficiency at 65–70°C and at pH 7.5. Substrate spectrum analysis was performed using various artificial chromogenic nitrophenyl‐linked sugars and natural di‐ and trisaccharides (Table 2) at 65°C and 37°C, respectively. The highest activity for chromogenic substrates was observed for *para‐*nitrophenyl‐β‐d‐galactopyranoside, with a *V*_max_ of 370 ± 43 U mg^‐1^ and a *K*_m_ value of 0.5 ± 0.3 mM, resulting in a *K_cat_* of 530.3 ± 87.1 s^−1^ and a *K*_cat_/*K*_m_ of 1060.6 ± 174.2 s^−1 ^mM^−1^ (Figure 1a). The enzymatic activity with *ortho*‐nitrophenyl‐β‐d‐galactopyranoside as substrate was approximately 10 times lower and with *para‐*nitrophenyl‐*N*‐acetyl‐β‐d‐galactosaminide only trace activity could be detected. The enzyme was inactive with all other nitrophenyl‐linked substrates (Table 2). The activity of Amuc_1686 was not influenced by the addition of 5 mM K^+^, Na^+^, Ca^2+^, Mg^2+^, Mn^2+^, Fe^3+^, La^3+^, and Mo^6+^ while addition of Ni^2+^, Co^2+^, and Ce^3+^ resulted in a decreased activity. Zn^2+^ or Cu^2+^ inhibited the reaction completely (not shown).

**Table 2:** Substrate spectrum of Amuc_1686 with different chromogenic and non‐chromogenic substrates

Chromogenic substrate^a^	Activity (U mg^-1^)	Non‐chromogenic substrate^b^	Cleavage
4‐Nitrophenyl‐β‐d‐glucopyranoside	<0.1	Sucrose (glucose‐ α, β ‐1,2‐fructose)	No
4‐Nitrophenyl‐α‐d‐glucopyranoside	<0.1	Trehalose (glucose‐ α, α ‐1,1‐glucose)	No
4‐Nitrophenyl‐β‐d‐glucuronide	<0.1	Melibiose (galactose‐ α‐1,6‐glucose)	No
2‐Nitrophenyl‐β‐d‐galactopyranoside	35 ± 4	Xylobiose (xylose‐ β‐1,4‐xylose)	No
4‐Nitrophenyl‐β‐d‐galactopyranoside	370 ± 43	Maltose (glucose‐ α‐1,4‐glucose)	No
4‐Nitrophenyl‐β‐d‐xylopyranoside	<0.1	Lactose (galactose‐β‐1,4‐glucose)	No
4‐Nitrophenyl‐*N*‐acetyl‐β‐d‐galactosaminide	0.2 ± 0.1	LacNAc (galactose‐β‐1,4‐*N*‐acetyl‐d‐glucosamine)	No
4‐Nitrophenyl‐*N*‐acetyl‐β‐d‐glucosaminide	<0.1	Galacto‐*N*‐biose (galactose‐β‐1,3‐*N*‐acetyl‐d‐galactosamine)	Yes
4‐Nitrophenyl‐β‐l‐fucopyranoside	<0.1	Raffinose (galactose‐α‐1,6‐glucose‐β‐1,2‐fructose)	No
4‐Nitrophenyl‐α‐l‐fucopyranoside	<0.1		
4‐Nitrophenyl‐α‐d‐arabinopyranoside	<0.1	*N*‐acetyl‐d‐glucosamine‐β‐1,3‐*N*‐acetyl‐d‐galactosamine	No
4‐Nitrophenyl‐α‐d‐galactopyranoside	<0.1		
4‐Nitrophenyl‐β‐galacto‐*N*‐bioside	<0.1	2′Fucosyllactose (fucose‐α‐1,2‐galactose‐β‐1,4‐glucose)	No
4‐Nitrophenyl‐*N*‐acetyl‐β‐d‐galactopyranoside	<0.1		

a Assays were performed in 50 mM combined buffer pH 7.5 at 65°C with a substrate concentration of 5 mM and product formation was analyzed photometrically.

b Assays were performed in 50 mM combined buffer pH 7.5 at 37°C with a substrate concentration of 5 mM and product formation was analyzed via HPLC.

**Figure 2:**
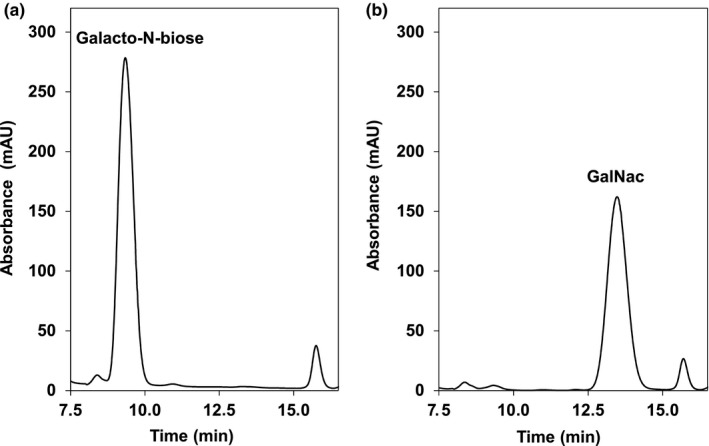
Enzymatic activity of Amuc_1686 with galacto‐*N*‐biose as Substrate. Combined puffer (150 µl) pH 7.5 containing 35 mM galacto‐*N*‐biose was incubated with 2.5 µg enzyme for 6 hr at 37°C. Substrate and products were analyzed via HPLC UV detection after an incubation time of 0 hr (a) and 6 hr (b). After 6 hr, more than 98% of galacto‐*N*‐biose (a) was hydrolyzed into GalNAc (b). Peak at 15.75 min = internal acetate standard. The experiment was conducted in triplicate using different protein preparations. One representative experiment is shown

Hydrolytic activity of Amuc_1686 with natural saccharides at 37°C was detected for galacto‐*N*‐biose, consisting of galactose that is β‐1,3 glycosidic bound to *N*‐acetyl‐d‐galactosamine (GalNAc). Examination of product formation revealed a cleavage of approximately 98% galacto‐*N*‐biose within 6 hr with an activity of 7.6 ± 0.5 U mg^‐1^ (Table 2, Figure 2).

### 3.2 Discovery of a discoidin domain in Amuc_1686

**Figure 3:**
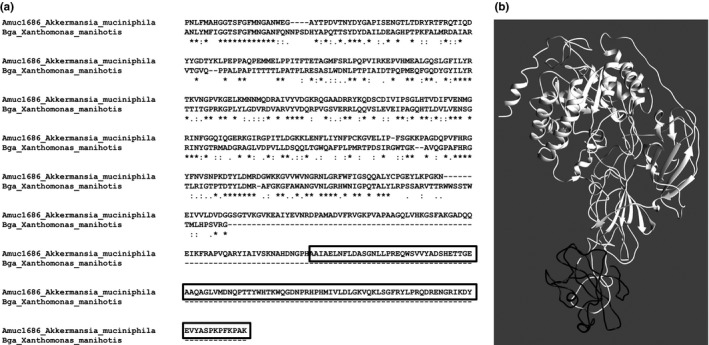
Alignment of Bga and Amuc_1686 and proposed structure of Amuc_1686. The amino acid sequence of Amuc_1686 and Bga from *Xanthomonas manihotis*were aligned (only partly shown), indicating the missing C‐terminal domain in Bga and the discoidin domain of Amuc_1686 (black framed, a). Phyre2 modeling of Amuc_1686 (b). White, catalytic domain; black, discoidin domain

In comparison to its closest homolog, the β‐galactosidase Bga from *X. manihotis*(Taron et al., 1995), Amuc_1686 possesses an additional C‐ terminal domain (Figure 3). Further analysis with the bioinformatic tools Pfam (Finn et al., 2016) and Prosite (Hulo et al., 2008; Sigrist et al., 2013) revealed that this part of the enzyme represents a truncated discoidin domain, also known as coagulation factor type C F5/8 domain or FA58C domain (Baumgartner, Hofmann, Chiquet‐Ehrismann, & Bucher, 1998; Foster, Fulcher, Houghten, & Zimmerman, 1990; Ortel et al., 1994). Discoidin domains are found in blood coagulation factors 5 and 8 as a twice‐repeated C‐terminal domain of approximately 150 amino acids. These domains promote binding to specific phospholipids on cell surfaces of, for example, platelets or endothelial cells. It is known that in the case of coagulation factor 8 this domain is crucial for enzyme activity and phosphatidylserine‐binding (Foster et al., 1990; Kane & Davie, 1988). To analyze the relative position of the discoidin domain in Amuc_1686, Phyre2 (Kelley, Mezulis, Yates, Wass, & Sternberg, 2015) and Chimera (Petersen et al., 2011) were used to create a tentative model of Amuc_1686. The predicted tertiary structure of Amuc_1686 showed a structural separation of the C‐terminal discoidin domain from the rest of the enzyme, which indicated a putative regulatory or interacting function of this domain.

NCBI BLASTp analysis of the discoidin domain of Amuc_1686 using the Swissprot database showed similarities with a sialidase from *Micromonospora viridifaciens*(NedA), a *N*‐acetyl‐beta‐hexosaminidase from *Clostridium perfringens*ATCC 13124 (NagJ), and a hyaluronoglucosaminidase from *C. perfringens* (NagH) (Ficko‐Blean & Boraston, 2006; Pathak, Dorfmueller, Borodkin, & Aalten, 2008; Rao et al., 2006). The average length of the discoidin domain of the three closest hits NagH, NagJ, and NedA was 130 ± 3 amino acids and for all three domains of these proteins carbohydrate binding functions were described. However, the discoidin domain of Amuc_1686 had only a length of 79 amino acids. An alignment with ClustalOmega was performed which revealed that in case of Amuc_1686 the C‐terminal part of the discoidin domain is missing.

### 3.3 The influence of different phospholipids on the enzymatic activity of Amuc_1686 and construction of a mutant enzyme of Amuc_1686 without C terminal discoidin domain

**Figure 4:**
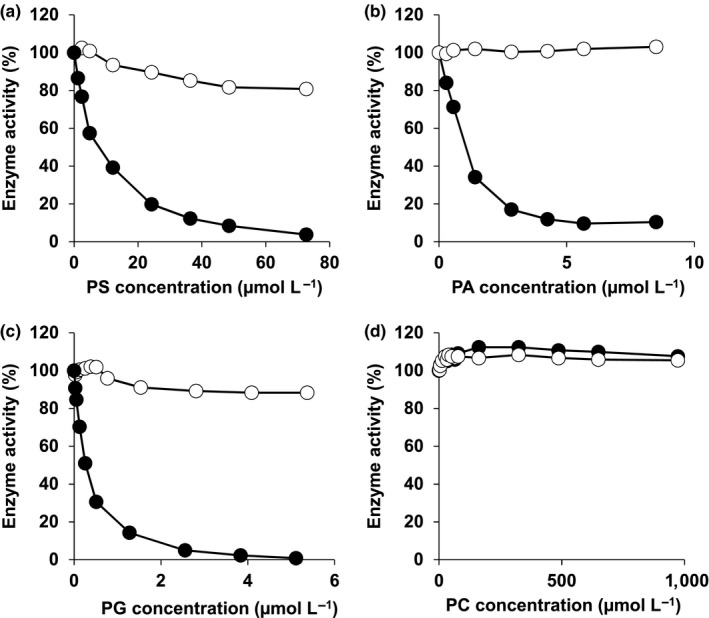
Analysis of the influence of an increasing concentration of phosphatidylserine (a), phosphatidic acid (b), phosphatidylglycerol (c) and phosphatidylcholine (d) on the enzymatic activity of Amuc_1686 (●) and Amuc_1686_short (○). Control experiments with methanol with an equimolar final concentration had no negative influence on the enzymatic activity. The reaction assays contained 1 µg Amuc_1686, 5 mM *para‐*nitrophenyl‐β‐d‐galactopyranoside and were performed in a combined buffer (50 mM) at pH 7.5 and at a temperature of 37°C. The activity was measured photometrically at 420 nm. The experiments were conducted at least in triplicates using three different Amuc_1686 preparations. One representative experiment is shown. The average specific activity of Amuc_1686 was 20.2 ± 2.5 U mg^-1^ protein (100%)

It is known that discoidin domains, for example, found in the carboxylterminus of blood coagulation factors 5 and 8, can promote binding to cell surface phospholipids such as phosphatidylserine and are responsible for enzyme activity (Foster et al., 1990; Kane & Davie, 1988). Thus, a possible interaction of the discoidin domain of Amuc_1686 with phospholipids was contemplated. For the analysis of the influence of phospholipids on the activity, enzyme reactions were performed with Amuc_1686 under standard conditions using *para‐*nitrophenyl‐β‐d‐galactopyranoside as substrate and the change of absorbance at a wavelength of 405 nm was recorded. After 30 s different amounts of phosphatidylserine (PS), phosphatidylglycerol (PG), phosphatidic acid (PA), phosphatidylcholine (PC) or phosphatidylethanolamine (PE) dissolved in methanol were added to the assay and the inhibiting effect of the phospholipid was analyzed. PC (Figure 4d) and PE (not shown) exhibited no inhibitory effect up to a final concentration of 1 mmol L^‐1^ in the enzymatic assay containing 1 µg Amuc_1686. However, PS, PA and PG had a strong inhibitory impact on Amuc_1686 (Figure 4a–c), indicated by IC_50_ values of 29.3 ± 1.5, 4.3 ± 0.2 and 1 ± 0.1 µmol L^‐1^, respectively. These values correspond to the *p*IC_50_ values 4.53 (PS), 5.37 (PA) and 6 (PG). Additionally, the potential inhibiting influence of lysophosphatidic acid (LPA) was investigated, showing an IC_50_ value of 13.6 ± 10.6 µmol L^‐1^ which corresponds to a *p*IC_50_ value of 4.87 (not shown). Control experiments using pure methanol did not reveal a negative influence on the enzymatic activity.

To answer the question whether the C terminus containing the discoidin domain is responsible for the phospholipid induced inhibition of Amuc_1686, a shortened variant of Amuc_1686 was constructed (Amuc_1686_short), lacking the last 107 amino acids. For inhibition experiments, 1 µg of Amuc_1686_short was applied to a standard assay that was identically performed as with Amuc_1686. When directly compared with Amuc_1686, the shortened variant exhibited a slightly decreased enzymatic activity but showed no significant inhibitory effect after addition of PS, PG and PA in the same concentration as applied to the assay containing the full‐length enzyme (Figure 4).

In order to analyze whether the hydrophilic head groups of the phospholipids have an influence on enzyme activity, l‐serine, glycerol, choline and ethanolamine were added to the standard assay up to a final concentration of 3 mmol L^‐1^. Under these conditions, no inhibition was observed. To identify the type of inhibition, the *K*_m_ value for *para‐*nitrophenyl‐β‐d‐galactopyranoside in the presence of 0.25 µmol L^‐1^ PG was determined. As the measured values were in the same range as without inhibitor addition, we concluded a non‐competitive type of inhibitory effect of the phospholipids on Amuc_1686.

## 4 DISCUSSION

The Gram‐negative gut bacterium *A. muciniphila* is able to utilize the mucus layer that covers colonic epithelial cells in the human large intestine. Mucin is used by this organism as carbon and nitrogen source which has an ecological advantage due to limitation of free carbon sources in this specific gut region (Derrien et al., 2008; Salyers et al., 1977). The degradation of the highly complex mammalian mucin glycan structures involves microbial cooperative action and a set of differently specialized GH (Crost et al., 2016; Derrien et al., 2004; Willis et al., 1996). Among other enzymes, especially β‐galactosidases play a major role in the efficient degradation of the oligosaccharide chains of mucins (Crost et al., 2016). Therefore, the characterization of the β‐galactosidase Amuc_1686 was done to get more insight into the mucin‐degrading mechanisms of *A. muciniphila*.

The only hydrolytic activity of Amuc_1686 with a non‐chromogenic substrate was detected with Galβ1–3GalNAc (Table 1). Therefore, we conclude that this type of glycosidic bond (also known as T antigen) is the target structure for the enzyme within the oligosaccharide chains of mucin. Galβ1–3GalNAc can be found as the central molecule within mucin core structure type 1 [Galβ1–3GalNAc‐*O*‐S/T] or type 2 [Galβ1–3(GlcNAcβ1–6)GalNAc‐*O*‐S/T] mucins. In the human transversal and descending colon, the natural habitat of *A. muciniphila*, the major part of mucin oligosaccharide structures is based on core structure type 3 [GlcNAcβ1–3GalNAc‐*O*‐S/T] or type 4 [GlcNAc1–3(GlcNAcβ1–6)GalNAc‐*O*‐S/T] (Holmén Larsson, Thomsson, Rodríguez‐Piñeiro, Karlsson, & Hansson, 2013; Robbe, Capon, Coddeville, & Michalski, 2004). However, more than half of core 3‐like structures exhibit galactose units, that are connected to the *N*‐acetyl‐d‐galactosamine residues [Galβ1‐(3/4)GlcNAcβ1–3GalNAc‐*O*‐S/T] (Robbe et al., 2004) by β1–3‐ or β1–4 glycosidic bonds. Additionally, it is known that the mucus of the sigmoidal colon is mainly made up by the gel‐forming MUC2 mucins, which contain primarily core type 3 structures as well (Etzold & Juge, 2014). The prevalence of these types of bonds in the human gut exhibits the necessity of specific extracellular β‐galactosidases such as Amuc_1686 for an efficient oligosaccharide cleavage.

Analysis of the primary structure of Amuc_1686 revealed a unique C‐terminal domain which can be bioinformatically classified as discoidin domain (Figure 2). In eukaryotic proteins, discoidin domains are widely distributed. Enzymes containing this domain exhibit various molecular functions such as the promotion of cell aggregation, blood coagulation, cell adhesion, cell recognition and cell‐cell interactions (Baumgartner et al., 1998; Sauer et al., 1997; Villoutreix & Miteva, 2016). During these events the discoidin domain itself is responsible for integrin receptor binding, phospholipid binding or carbohydrate chain binding (Borisenko, Iverson, Ahlberg, Kagan, & Fadeel, 2004; Hidai et al., 1998; Poole, Firtel, Lamar, & Rowekamp, 1981). In this work, we could show a strong inhibitory regulation of Amuc_1686 from *A. muciniphila* by the anionic phospholipids PA, PG, PS and LPA. It became evident that the C‐terminal discoidin domain of the enzyme is responsible for this regulatory effect because the shortened variant of Amuc_1686, missing the discoidin domain, showed no significant inhibition by these lipids.

**Figure 5:**
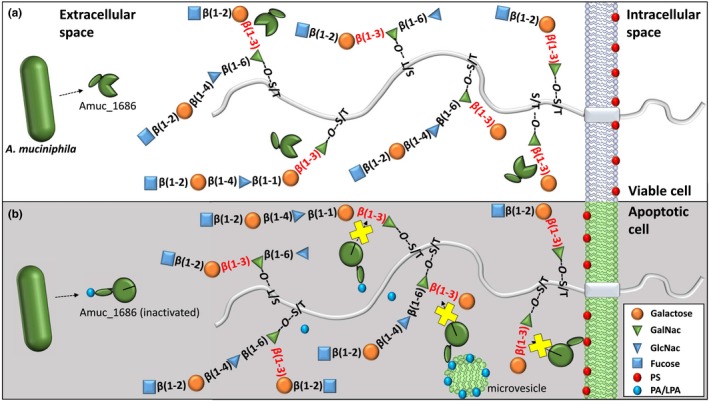
Target structures of extracellular β‐galactosidase Amuc_1686 on viable (a) and apoptotic epithelial cells (b). The Galβ1–3GalNac target structures of Amuc_1686 are shown in red. The inability to hydrolyze these structures by inactivated Amuc_1686 is indicated by a yellow cross (b). Active and inactive Amuc_1686 proteins are indicated by open green circles and closed green circles, respectively. Inhibiting phospholipids (phosphatidylserine = red, phosphatidic acid/lysophosphatidic acid = blue) occur in apoptotic or wounded tissues on the surface of the cells or shed microvesicles

In eukaryotic cells, enzyme inhibition by phospholipids was previously described (Stace & Ktistakis, 2006). The γ isoform of the human protein phosphatase‐1 catalytic subunit (PP1cγ) is a high‐affinity target of the bioactive lipid second messenger PA which inhibits the enzyme non‐competitively and dose dependently with an IC_50_of 15 nM (Jones & Hannun, 2002). Moreover, PS and PA were shown to inhibit the Ca^2+^‐ATPase of the sarcoplasmic reticulum (Dalton et al., 1998). In addition, examples for proteins able to interact with all three phospholipids PS, PA and PG are known from literature, for instance the high mobility group box 1 protein This protein is a mediator of inflammation which is secreted by monocytes, macrophages and dendritic cells and plays a key role in late phase of injury (He et al., 2011; Wang et al., 1999). Here we present another example of a protein which is regulated by anionic phospholipids. To our knowledge, this is the first characterization of a prokaryotic hydrolase that is strongly inhibited by phospholipids. Due to the strong and obvious beneficial host‐microbe relationship between *A. muciniphila* und the human intestinal tract, this regulatory system might reflect a mechanism which allows this mutualistic relationship (Figure 5).

It is known that *A. muciniphila*positively influences and promotes wound healing and tissue regeneration (Alam et al., 2016). Due to its metabolism, which includes the degradation of intestinal mucins that act as innate host defense and protective barrier to infections, the gut health‐promoting properties seem to be contradictory (Dharmani, Srivastava, Kissoon‐Singh, & Chadee, 2009; Linden, Sutton, Karlsson, Korolik, & McGuckin, 2008). However, when apoptosis due to infection or tissue damage of epithelial cells is induced, mucin‐like structures play a key role in many events crucial for immune response, cell renewal and tissue regeneration (Figure 5). One of the first events during programed cell death in epithelial cells is the exposure of PS, which constitutes 5%–10% of total cellular lipid, on the outer leaflet of the membrane (Stace & Ktistakis, 2006; Yamaji‐Hasegawa & Tsujimoto, 2006). The normal asymmetrical architecture and the rapid externalization of PS is one of the most important “eat me” signals in the clearance process. In addition, specific mucin‐like structures on the epithelial cell surfaces are necessary for a proper cell‐cell interaction with dendritic cells and macrophages (Ravichandran, 2010). In view of the mostly mutualistic relationship between *A. muciniphila* and its host, a regulation of the extracellular mucin‐degrading enzyme Amuc_1686 on protein level is conceivable (Figure 5). An interaction of Amuc_1686 with exposed PS might lead to a down‐regulation of the hydrolytic activity in regions with increased numbers of apoptotic cells or wounded tissues, which would result in an unimpeded immune response and therefore an improved tissue regeneration and wound healing.

In addition to PS, a strong inhibitory regulation of Amuc_1686 was observed by the anionic phospholipids PA, PG and LPA. PA is known to serve as a lipid second messenger (McPhail et al., 1999; Stace & Ktistakis, 2006; Voelker, 1991) and is a central pro‐inflammatory mediator whose generation is initiated by inflammatory cytokines such as tumor necrosis factor‐α and interleukin‐1β (Greaves & Camp, 1988; Sturm, 2002). LPA as a derivative of PA can be formed extracellularly by the action of inflammatory type II phospholipase A2 or phospholipases from pathogens by hydrolysis of PA from membrane surfaces in intestinal epithelial cells. LPA shows pro‐inflammatory properties similar to PA (Fourcade et al., 1995; Moolenaar, 2000). In wounded tissues, PA and LPA can be present nearby apoptotic stressed epithelial cells, occurring on the surface of small shed microvesicles (Fourcade et al., 1995). Thereby PA and LPA could act as sensible inhibitors of Amuc_1686 leading to the protection of the mucin layer in regions of the large intestine with tissue irritation, injury, or infection (Figure 5). An inhibitory effect on Amuc_1686 was not detected in the presence of PC and PE. These phospholipids are found in high abundance in membranes of viable eukaryotic cells, constituting about 40%–50% and 20%–50% of total phospholipids, respectively (Chaurio et al., 2009; Yamaji‐Hasegawa & Tsujimoto, 2006).

Another aspect is that carbohydrate chains of the cellular surface function as a signal for apoptotic cell removal (Beppu, Eda, Fujimaki, Hishiyama, & Kikugawa, 1996; Yamanaka, Eda, & Beppu, 2005). It is known that a transient accumulation of the mucin‐like major sialoglycoprotein CD43 (also known as leukosialin) occurs on membranes of apoptotic cells. The binding of CD43 results in a condensation of the carbohydrate chains on the cellular surface of the apoptotic cell leading to the formation of target structures for the binding of macrophages (Eda, Yamanaka, & Beppu, 2004; Yamanaka et al., 2005). In the mucin‐like structures of CD43, linkages from the type Galβ1–3GalNAc are very common (Piller, Deist, Weinberg, Parkman, & Fukuda, 1991). Interaction of exposed PS with the discoidin domain of Amuc_1686 would inhibit enzyme activity (Figure 5) avoiding a degradation of this silyl polylactosaminyl saccharide ligands. Such a regulation would be highly beneficial for the host, as a degradation of these oligosaccharide chains would lead to a deterioration of macrophage recognition and therefore a worse clearance and tissue regeneration. Moreover, a slowed removal of apoptotic cells would cause further inflammation and autoimmune responses against intracellular antigens released from dying cells (Kobayashi et al., 2007; Savill & Fadok, 2000).

A further example for a mucin‐like structure involved in immune response is the glycoprotein lactadherin which recognizes and binds to PS by a discoidin domain. This protein contains mucin‐like glycosidic structures, enhances phagocytosis and is crucial for repair of intestinal epithelium (Bu et al., 2007). Additionally, T cell immunoglobulin mucin proteins 1 and 4 (TIM‐1, TIM‐4) expressed on mammalian macrophages and dendritic cells specifically bind PS on the surface of apoptotic cells and mediate T cell activation and apoptotic cell uptake. Structurally TIM 1 and 4 are glycoproteins as well, exhibiting important mucin‐like glycosidic structures (Kobayashi et al., 2007). Hence, the inhibition of Amuc_1686 by PS would lead to a protection of mucin‐like structures found in important compounds of the immune system.

In summary, Amuc_1686 can be classified as a phospholipid‐regulated enzyme that might not attack apoptotic epithelial cells to ensure tissue regeneration and to avoid a disorder of the clearance process. This mutualistic behavior would support health of the host but also implicates that substrate for growth of *A. muciniphila* is limited from damaged areas of the large intestine. However, further experiments have to be done in the future to verify or to refute the proposed hypothesis.

## CONFLICT OF INTEREST

There is no conflict of interest for all authors.

## AUTHORS CONTRIBUTION

Uwe Deppenmeier and Konrad Kosciow conceived and designed the research. Konrad Kosciow performed the experiments and wrote the initial manuscript draft. Uwe Deppenmeier supervised the research and revised the manuscript draft.

## ETHICS STATEMENT

None required.

## Data Availability

Data available upon request from the authors.

## References

[mbo3796-bib-0001] Alam, A. , Leoni, G. , Quiros, M. , Wu, H. , Desai, C. , Nishio, H. , … Neish, A. S. (2016). The microenvironment of injured murine gut elicits a local pro‐restitutive microbiota. NatureMicrobiology, 1(2), 15021 10.1038/nmicrobiol.2015.21 PMC5076466 27571978

[mbo3796-bib-0002] Baumgartner, S. , Hofmann, K. , Chiquet‐Ehrismann, R. , & Bucher, P. (1998). The discoidin domain family revisited: New members from prokaryotes and a homology‐based fold prediction. Protein Science, 7(7), 1626–1631. 10.1002/pro.5560070717 9684896 PMC2144056

[mbo3796-bib-0003] Beppu, M. , Eda, S. , Fujimaki, M. , Hishiyama, E. , & Kikugawa, K. (1996). Recognition of poly‐N‐acetyllactosaminyl saccharide chains on iron‐oxidized erythrocytes by human monocytic leukemia cell line THP‐1 differentiated into macrophages. Biological and Pharmaceutical Bulletin, 19(2), 188–194. 10.1248/bpb.19.188 8850303

[mbo3796-bib-0004] Blum, H. , Beier, H. , & Gross, H. J. (1987). Improved silver staining of plant proteins, RNA and DNA in polyacrylamide gels. Electrophoresis, 8(2), 93–99. 10.1002/elps.1150080203

[mbo3796-bib-0005] Borisenko, G. G. , Iverson, S. L. , Ahlberg, S. , Kagan, V. E. , & Fadeel, B. (2004). Milk fat globule epidermal growth factor 8 (MFG‐E8) binds to oxidized phosphatidylserine: Implications for macrophage clearance of apoptotic cells. Cell Death and Differentiation, 11(8), 943 10.1038/sj.cdd.4401421 15031725

[mbo3796-bib-0006] Bu, H.‐F. , Zuo, X.‐L. , Wang, X. , Ensslin, M. A. , Koti, V. , Hsueh, W. , … Tan, X.‐D. (2007). Milk fat globule–EGF factor 8/lactadherin plays a crucial role in maintenance and repair of murine intestinal epithelium. The Journal of Clinical Investigation, 117(12), 3673–3683. 10.1172/JCI31841 18008006 PMC2075476

[mbo3796-bib-0007] Chaurio, R. A. , Janko, C. , Muñoz, L. E. , Frey, B. , Herrmann, M. , & Gaipl, U. S. (2009). Phospholipids: Key players in apoptosis and immune regulation. Molecules, 14(12), 4892–4914. 10.3390/molecules14124892 20032867 PMC6255253

[mbo3796-bib-0008] Collado, M. C. , Derrien, M. , Isolauri, E. , de Vos, W. M. , & Salminen, S. (2007). Intestinal integrity and *Akkermansia muciniphila*, a mucin‐degrading member of the intestinal microbiota present in infants, adults, and the elderly. Applied and Environmental Microbiology, 73(23), 7767–7770. 10.1128/AEM.01477-07 17933936 PMC2168041

[mbo3796-bib-0009] Crost, E. H. , Tailford, L. E. , Monestier, M. , Swarbreck, D. , Henrissat, B. , Crossman, L. C. , & Juge, N. (2016). The mucin‐degradation strategy of *Ruminococcus gnavus*: The importance of intramolecular trans‐sialidases. Gut Microbes, 7(4), 302–312. 27223845 10.1080/19490976.2016.1186334 PMC4988440

[mbo3796-bib-0010] Dalton, A. K. , East, J. M. , Sanjay, M. , Oliver, S. , Starling, P. A. , & Lee, G. A. (1998). Interaction of phosphatidic acid and phosphatidylserine with the Ca2+‐ATPase of sarcoplasmic reticulum and the mechanism of inhibition. Biochemical Journal, 329(3), 637–646. 10.1042/bj3290637 9445393 PMC1219087

[mbo3796-bib-0011] Derrien, M. , Collado, M. C. , Ben‐Amor, K. , Salminen, S. , & de Vos, W. M. (2008). The Mucin degrader *Akkermansia muciniphila* is an abundant resident of the human intestinal tract. Applied and Environmental Microbiology, 74(5), 1646–1648. 10.1128/AEM.01226-07 18083887 PMC2258631

[mbo3796-bib-0012] Derrien, M. , van Baarlen, P. , Hooiveld, G. , Norin, E. , Müller, M. , & de Vos, W. M. (2011). Modulation of Mucosal Immune Response, Tolerance, and Proliferation in Mice Colonized by the Mucin‐Degrader *Akkermansia muciniphila* . Frontiers in Microbiology, 2, 166 10.3389/fmicb.2011.00166 21904534 PMC3153965

[mbo3796-bib-0013] Derrien, M. , Vaughan, E. E. , Plugge, C. M. , & de Vos, W. M. (2004). *Akkermansia muciniphila* gen. nov., sp. nov., a human intestinal mucin‐degrading bacterium. International Journal of Systematic and Evolutionary Microbiology, 54(5), 1469–1476. 10.1099/ijs.0.02873-0 15388697

[mbo3796-bib-0014] Dharmani, P. , Srivastava, V. , Kissoon‐Singh, V. , & Chadee, K. (2009). Role of intestinal mucins in innate host defense mechanisms against pathogens. Journal of Innate Immunity, 1(2), 123–135. 10.1159/000163037 20375571 PMC7312850

[mbo3796-bib-0015] Eda, S. , Yamanaka, M. , & Beppu, M. (2004). Carbohydrate‐mediated phagocytic recognition of early apoptotic cells undergoing transient capping of CD43 glycoprotein. Journal of Biological Chemistry, 279(7), 5967–5974. 10.1074/jbc.M310805200 14613931

[mbo3796-bib-0016] Etzold, S. , & Juge, N. (2014). Structural insights into bacterial recognition of intestinal mucins. Current Opinion in Structural Biology, 28, 23–31. 10.1016/j.sbi.2014.07.002 25106027

[mbo3796-bib-0017] Everard, A. , Belzer, C. , Geurts, L. , Ouwerkerk, J. P. , Druart, C. , Bindels, L. B. , … Cani, P. D. (2013). Cross‐talk between *Akkermansia muciniphila* and intestinal epithelium controls diet‐induced obesity. Proceedings of the National Academy of Sciences, 110(22), 9066–9071. 10.1073/pnas.1219451110 PMC3670398 23671105

[mbo3796-bib-0018] Ficko‐Blean, E. , & Boraston, A. B. (2006). The interaction of a carbohydrate‐binding module from a *Clostridium perfringens* N‐acetyl‐beta‐hexosaminidase with its carbohydrate receptor. Journal of Biological Chemistry, 281, 37748–37757. 10.1074/jbc.m606126200 16990278

[mbo3796-bib-0019] Finn, R. D. , Coggill, P. , Eberhardt, R. Y. , Eddy, S. R. , Mistry, J. , Mitchell, A. L. , … Bateman, A. (2016). The Pfam protein families database: Towards a more sustainable future. Nucleic Acids Research, 44, 279–285. 10.1093/nar/gkv1344 PMC4702930 26673716

[mbo3796-bib-0020] Foster, P. A. , Fulcher, C. A. , Houghten, R. A. , & Zimmerman, T. S. (1990). Synthetic factor VIII peptides with amino acid sequences contained within the C2 domain of factor VIII inhibit factor VIII binding to phosphatidylserine. Blood, 75(10), 1999–2004. 2110840

[mbo3796-bib-0021] Fourcade, O. , Simon, M.‐F. , Viodé, C. , Rugani, N. , Leballe, F. , Ragab, A. , … Chap, H. (1995). Secretory phospholipase A2 generates the novel lipid mediator lysophosphatidic acid in membrane microvesicles shed from activated cells. Cell, 80(6), 919–927. 10.1016/0092-8674(95)90295-3 7697722

[mbo3796-bib-0022] Ganesh, B. P. , Klopfleisch, R. , Loh, G. , & Blaut, M. (2013). Commensal *Akkermansia muciniphila* exacerbates gut inflammation in *Salmonella typhimurium*‐infected gnotobiotic mice. PLoS ONE, 8(9), e74963 10.1371/journal.pone.0074963 24040367 PMC3769299

[mbo3796-bib-0023] Greaves, M. W. , & Camp, R. D. (1988). Prostaglandins, leukotrienes, phospholipase, platelet activating factor, and cytokines: An integrated approach to inflammation of human skin. Archives of Dermatological Research, 280, S33–41. 2841909

[mbo3796-bib-0024] He, M. , Kubo, H. , Morimoto, K. , Fujino, N. , Suzuki, T. , Takahasi, T. , … Yamamoto, H. (2011). Receptor for advanced glycation end products binds to phosphatidylserine and assists in the clearance of apoptotic cells. EMBO Reports, 12(4), 358–364. 10.1038/embor.2011.28 21399623 PMC3077249

[mbo3796-bib-0025] Hidai, C. , Zupancic, T. , Penta, K. , Mikhail, A. , Kawana, M. , Quertermous, E. E. , … Quertermous, T. (1998). Cloning and characterization of developmental endothelial locus‐1: An embryonic endothelial cell protein that binds the alpha vbeta 3 integrin receptor. Genes and Development, 12(1), 21–33. 10.1101/gad.12.1.21 9420328 PMC529342

[mbo3796-bib-0026] Holmén Larsson, J. M. , Thomsson, K. A. , Rodríguez‐Piñeiro, A. M. , Karlsson, H. , & Hansson, G. C. (2013). Studies of mucus in mouse stomach, small intestine, and colon. III. Gastrointestinal Muc5ac and Muc2 mucin O‐glycan patterns reveal a regiospecific distribution. American Journal of Physiology ‐ Gastrointestinal and Liver Physiology, 305(5), 357–363. 10.1152/ajpgi.00048.2013 PMC3761246 23832516

[mbo3796-bib-0027] Hoskins, L. C. , & Boulding, E. T. (1981). Mucin degradation in human colon ecosystems. Evidence for the existence and role of bacterial subpopulations producing glycosidases as extracellular enzymes. The Journal of Clinical Investigation, 67(1), 163–172. 10.1172/JCI110009 6161136 PMC371584

[mbo3796-bib-0028] Hulo, N. , Bairoch, A. , Bulliard, V. , Cerutti, L. , Cuche, B. A. , de Castro, E. , … Sigrist, C. J. (2008). The 20 years of PROSITE. Nucleic Acids Research, 36, 245–249. 10.1093/nar/gkm977 18003654 PMC2238851

[mbo3796-bib-0029] Jones, J. A. , & Hannun, Y. A. (2002). Tight‐binding inhibition of protein phosphatase‐1 by phosphatidic acid: Specificity of inhibition by the phospholipid. Journal of Biological Chemistry, 277(18), 15530–15538. 10.1074/jbc.M111555200 11856740

[mbo3796-bib-0030] Käll, L. , Krogh, A. , & Sonnhammer, E. L. L. (2004). A combined transmembrane topology and signal peptide prediction method. Journal of Molecular Biology, 338(5), 1027–1036. 10.1016/j.jmb.2004.03.016 15111065

[mbo3796-bib-0031] Kane, W. H. , & Davie, E. W. (1988). Blood coagulation factors V and VIII: Structural and functional similarities and their relationship to hemorrhagic and thrombotic disorders. Blood, 71(3), 539–555. 3125864

[mbo3796-bib-0032] Kang, C. S. , Ban, M. , Choi, E. J. , Moon, H. G. , Jeon, J. S. , Kim, D. K. , … Kim, Y. K. (2013). Extracellular vesicles derived from gut microbiota, especially *Akkermansia muciniphila*, protect the progression of dextran sulfate sodium‐induced colitis. PLoS ONE, 8(10), e76520 10.1371/journal.pone.0076520 24204633 PMC3811976

[mbo3796-bib-0033] Kelley, L. A. , Mezulis, S. , Yates, C. M. , Wass, M. N. , & Sternberg, M. J. E. (2015). The Phyre2 web portal for protein modeling, prediction and analysis. Nature Protocols, 10(6), 845–858. 10.1038/nprot.2015.053 25950237 PMC5298202

[mbo3796-bib-0034] Kobayashi, N. , Karisola, P. , Peña‐Cruz, V. , Dorfman, D. M. , Jinushi, M. , Umetsu, S. E. , … Freeman, G. J. (2007). TIM‐1 and TIM‐4 glycoproteins bind phosphatidylserine and mediate uptake of apoptotic cells. Immunity, 27(6), 927–940. 10.1016/j.immuni.2007.11.011 18082433 PMC2757006

[mbo3796-bib-0035] Kosciow, K. , Domin, C. , Schweiger, P. , & Deppenmeier, U. (2016). Extracellular targeting of an active endoxylanase by a TolB negative mutant of Gluconobacter oxydans. Journal of Industrial Microbiology and Biotechnology, 43(7), 989–999. 10.1007/s10295-016-1770-6 27097633

[mbo3796-bib-0036] Laemmli, U. K. (1970). Cleavage of structural proteins during the assembly of the head of bacteriophage T4. Nature, 227(5259), 680–685. 10.1038/227680a0 5432063

[mbo3796-bib-0037] Linden, S. K. , Sutton, P. , Karlsson, N. G. , Korolik, V. , & McGuckin, M. A. (2008). Mucins in the mucosal barrier to infection. Mucosal Immunology, 1(3), 183–197. 10.1038/mi.2008.5 19079178 PMC7100821

[mbo3796-bib-0038] McPhail, L. C. , Waite, K. A. , Regier, D. S. , Nixon, J. B. , Qualliotine‐Mann, D. , Zhang, W.‐X. , … Sergeant, S. (1999). A novel protein kinase target for the lipid second messenger phosphatidic acid. *Biochimica et* *Biophysica Acta (BBA)‐Molecular and Cell Biology of* . Lipids, 1439(2), 277–290. 10.1016/S1388-1981(99)00100-6 10425401

[mbo3796-bib-0039] Miller, J. H. (1972). Experiments in molecular genetics. Cold Spring Harbor, NY: Cold Spring Harbor Laboratory.

[mbo3796-bib-0040] Moolenaar, W. H. (2000). Development of Our Current Understanding of Bioactive Lysophospholipids. Annals of the New York Academy of Sciences, 905(1), 1–10. 10.1111/j.1749-6632.2000.tb06532.x 10818436

[mbo3796-bib-0041] Nielsen, H. , Engelbrecht, J. , Brunak, S. , & von Heijne, G. (1997). Identification of prokaryotic and eukaryotic signal peptides and prediction of their cleavage sites. Protein Engineering Design and Selection, 10(1), 1–6. 10.1093/protein/10.1.1 9051728

[mbo3796-bib-0042] Ortel, T. L. , Quinn‐Allen, M. A. , Keller, F. G. , Peterson, J. A. , Larocca, D. , & Kane, W. H. (1994). Localization of functionally important epitopes within the second C‐type domain of coagulation factor V using recombinant chimeras. Journal of Biological Chemistry, 269(22), 15898–15905. 7515064

[mbo3796-bib-0043] Pathak, S. , Dorfmueller, H. C. , Borodkin, V. S. , & van Aalten, D. M. F. (2008). Chemical dissection of the link between streptozotocin, O‐GlcNAc, and pancreatic cell death. Chemistry and Biology, 15(8), 799–807. 10.1016/j.chembiol.2008.06.010 18721751 PMC2568864

[mbo3796-bib-0044] Petersen, T. N. , Brunak, S. , von Heijne, G. , & Nielsen, H. (2011). SignalP 4.0: Discriminating signal peptides from transmembrane regions. NatureMethods, 8(10), 785–786. 10.1038/nmeth.1701 21959131

[mbo3796-bib-0045] Piller, F. , Le Deist, F. , Weinberg, K. I. , Parkman, R. , & Fukuda, M. (1991). Altered O‐glycan synthesis in lymphocytes from patients with Wiskott‐Aldrich syndrome. Journal of Experimental Medicine, 173(6), 1501–1510. 10.1084/jem.173.6.1501 2033371 PMC2190829

[mbo3796-bib-0046] Poole, S. , Firtel, R. A. , Lamar, E. , & Rowekamp, W. (1981). Sequence and expression of the discoidin I gene family in *Dictyostelium discoideum* . Journal of Molecular Biology, 153(2), 273–289. 10.1016/0022-2836(81)90278-3 6279874

[mbo3796-bib-0047] Rao, F. V. , Dorfmueller, H. C. , Villa, F. , Allwood, M. , Eggleston, I. M. , & van Aalten, D. M. F. (2006). Structural insights into the mechanism and inhibition of eukaryotic O‐GlcNAc hydrolysis. The EMBO Journal, 25(7), 1569–1578. 10.1038/sj.emboj.7601026 16541109 PMC1440316

[mbo3796-bib-0048] Ravichandran, K. S. (2010). Find‐me and eat‐me signals in apoptotic cell clearance: Progress and conundrums. Journal of Experimental Medicine, 207(9), 1807–1817. 10.1084/jem.20101157 20805564 PMC2931173

[mbo3796-bib-0049] Reunanen, J. , Kainulainen, V. , Huuskonen, L. , Ottman, N. , Belzer, C. , Huhtinen, H. , … Satokari, R. (2015). *Akkermansia muciniphila* Adheres to Enterocytes and Strengthens the Integrity of the Epithelial Cell Layer. Applied and Environmental Microbiology, 81(11), 3655–3662. 10.1128/AEM.04050-14 25795669 PMC4421065

[mbo3796-bib-0050] Robbe, C. , Capon, C. , Coddeville, B. , & Michalski, J.‐C. (2004). Structural diversity and specific distribution of O‐glycans in normal human mucins along the intestinal tract. The Biochemical Journal, 384(2), 307–316. 10.1042/BJ20040605 15361072 PMC1134114

[mbo3796-bib-0051] Salyers, A. A. , West, S. E. , Vercellotti, J. R. , & Wilkins, T. D. (1977). Fermentation of mucins and plant polysaccharides by anaerobic bacteria from the human colon. Applied and Environmental Microbiology, 34(5), 529–533. 563214 10.1128/aem.34.5.529-533.1977 PMC242695

[mbo3796-bib-0052] Sambrook, J. , Fritsch, E. F. , & Maniatis, T. (1989). Molecular cloning. A laboratory manual. Cold Spring Harbor, NY: Cold Spring Harbor Laboratory.

[mbo3796-bib-0053] Sauer, C. G. , Gehrig, A. , Warneke‐Wittstock, R. , Marquardt, A. , Ewing, C. C. , Gibson, A. , … Weber, B. H. (1997). Positional cloning of the gene associated with X‐linked juvenile retinoschisis. Nature Genetics, 17(2), 164–170. 10.1038/ng1097-164 9326935

[mbo3796-bib-0054] Savill, J. , & Fadok, V. (2000). Corpse clearance defines the meaning of cell death. Nature, 407(6805), 784–788. 10.1038/35037722 11048729

[mbo3796-bib-0055] Schneeberger, M. , Everard, A. , Gómez‐Valadés, A. G. , Matamoros, S. , Ramírez, S. , Delzenne, N. M. , … Cani, P. D. (2015). *Akkermansia muciniphila* inversely correlates with the onset of inflammation, altered adipose tissue metabolism and metabolic disorders during obesity in mice. Scientific Reports, 5(16643), 1–14. 10.1038/srep16643 PMC4643218 26563823

[mbo3796-bib-0056] Shin, N.‐R. , Lee, J.‐C. , Lee, H.‐Y. , Kim, M.‐S. , Whon, T. W. , Lee, M.‐S. , & Bae, J.‐W. (2013). An increase in the *Akkermansia*spp. population induced by metformin treatment improves glucose homeostasis in diet‐induced obese mice. Gut, 63(5), 727–735. 10.1136/gutjnl-2012-303839 23804561

[mbo3796-bib-0057] Sigrist, C. J. A. , de Castro, E. , Cerutti, L. , Cuche, B. A. , Hulo, N. , Bridge, A. , … Xenarios, I. (2013). New and continuing developments at PROSITE. Nucleic Acids Research, 41(1), 344–347. 10.1093/nar/gks1067 PMC3531220 23161676

[mbo3796-bib-0058] Stace, C. L. , & Ktistakis, N. T. (2006). Phosphatidic acid‐and phosphatidylserine‐binding proteins. *Biochimica et* *Biophysica Acta (BBA)‐Molecular and Cell Biology of* . Lipids, 1761(8), 913–926. 10.1016/j.bbalip.2006.03.006 16624617

[mbo3796-bib-0059] Sturm, A. (2002). Modulation of gastrointestinal wound repair and inflammation by phospholipids. *Biochimica et* *Biophysica Acta (BBA) ‐ Molecular and Cell Biology of* . Lipids, 1582(1), 282–288. 10.1016/S1388-1981(02)00182-8 12069839

[mbo3796-bib-0060] Taron, C. H. , Benner, J. S. , Hornstra, L. J. , & Guthrie, E. P. (1995). A novel β‐galactosidase gene isolated from the bacterium *Xanthomonas manihotis* exhibits strong homology to several eukaryotic β‐galactosidases. Glycobiology, 5(6), 603–610. 10.1093/glycob/5.6.603 8563148

[mbo3796-bib-0061] Villoutreix, B. O. , & Miteva, M. A. (2016). Discoidin Domains as Emerging Therapeutic Targets. Trends in Pharmacological Sciences, 37(8), 641–659. 10.1016/j.tips.2016.06.003 27372370

[mbo3796-bib-0062] Voelker, D. R. (1991). Organelle biogenesis and intracellular lipid transport in eukaryotes. Microbiological Reviews, 55(4), 543–560. 1779926 10.1128/mr.55.4.543-560.1991 PMC372837

[mbo3796-bib-0063] Wang, H. , Bloom, O. , Zhang, M. , Vishnubhakat, J. M. , Ombrellino, M. , Che, J. , … Tracey, K. J. (1999). HMG‐1 as a late mediator of endotoxin lethality in mice. Science, 285(5425), 248–251. 10.1126/science.285.5425.248 10398600

[mbo3796-bib-0064] Wang, L. , Christophersen, C. T. , Sorich, M. J. , Gerber, J. P. , Angley, M. T. , & Conlon, M. A. (2011). Low relative abundances of the mucolytic bacterium *Akkermansia muciniphila* and *Bifidobacterium* spp. in feces of children with autism. Applied and Environmental Microbiology, 77(18), 6718–6721. 10.1128/AEM.05212-11 21784919 PMC3187122

[mbo3796-bib-0065] Willis, C. L. , Cummings, J. H. , Neale, G. , & Gibson, G. R. (1996). In vitro effects of mucin fermentation on the growth of human colonic sulphate‐reducing bacteria. Anaerobe, 2(2), 117–122. 10.1006/anae.1996.0015

[mbo3796-bib-0066] Yamaji‐Hasegawa, A. , & Tsujimoto, M. (2006). Asymmetric distribution of phospholipids in biomembranes. Biological and Pharmaceutical Bulletin, 29(8), 1547–1553. 10.1248/bpb.29.1547 16880602

[mbo3796-bib-0067] Yamanaka, M. , Eda, S. , & Beppu, M. (2005). Carbohydrate chains and phosphatidylserine successively work as signals for apoptotic cell removal. Biochemical and Biophysical Research Communications, 328(1), 273–280. 10.1016/j.bbrc.2004.12.171 15670780

